# Evaluating cytology for the detection of invasive cervical cancer

**DOI:** 10.1111/cyt.12259

**Published:** 2015-06-30

**Authors:** R. Landy, A. Castanon, W. Hamilton, A. W. W. Lim, N. Dudding, A. Hollingworth, P. D. Sasieni

**Affiliations:** ^1^ Centre for Cancer PreventionWolfson Institute of Preventive MedicineBart's & The London School of MedicineQueen Mary University of LondonLondonUK; ^2^ College House, St Luke's CampusExeterUK; ^3^ Whipps Cross University, HospitalBarts Health NHS TrustLondonUK; ^4^ Cytology DepartmentSheffield Teaching Hospitals, NHS Foundation TrustSheffieldUK

**Keywords:** cervical cancer, cervical cytology, predictive value of tests, sensitivity, specificity, pap test

## Abstract

**Objective:**

To assess the sensitivity, the number needed to screen (NNS) and the positive predictive value (PPV) of cervical cytology for the diagnosis of cancer by age in a screening population.

**Methods:**

A retrospective cohort of women with invasive cervical cancer nested within a census of cervical cytology. All (*c*. 8 million) women aged 20–64 years with cervical cytology (excluding tests after an earlier abnormality). From April 2007 to March 2010, 3372 women had cervical cancer diagnosed within 12 months of such cytology in England. The sensitivity of cervical cytology to cancer, NNS to detect one cancer and predictive values of cytology were calculated for various ’referral‘ thresholds. These were calculated for ages 20–24, 25–34, 35–49 and 50–64 years.

**Results:**

The sensitivity of at least moderate dyskaryosis [equivalent to a high‐grade squamous intraepithelial lesion (HSIL) or worse] for cancer of 89.4% [95% confidence interval (CI) 88.3–90.4%] in women offered screening was independent of age. At all ages, women with borderline‐early recall or mild dyskaryosis on cytology (equivalent to ASC‐US and LSIL, respectively, in the Bethesda system) had a similar risk of cervical cancer to the risk in all women tested. The PPV of severe dyskaryosis/?invasive and ?glandular neoplasia cytology (equivalent to squamous cell carcinoma and adenocarcinoma/adenocarcinoma *in situ*, respectively, in the Bethesda System) were 34% and 12%, respectively; the PPV of severe dyskaryosis (HSIL: severe dysplasia) was 4%. The NNS was lowest when the incidence of cervical cancer was highest, at ages 25–39 years, but the proportion of those with abnormal cytology who have cancer was also lowest in younger women.

**Conclusions:**

The PPV of at least severe dyskaryosis (HSIL: severe dysplasia) for cancer was 4–10% of women aged 25–64 years, justifying a 2‐week referral to colposcopy and demonstrating the importance of failsafe monitoring for such patients. The sensitivity of cytology for cervical cancer was excellent across all age groups.

## Introduction

Cervical screening aims to prevent cervical cancer through the diagnosis and treatment of premalignant cervical lesions. Although screening can lead to the early diagnosis of invasive cervical cancer, which is not its primary goal, and the value of cytology to detect cancer (rather than pre‐cancerous lesions) has not been studied.

This paper considers the results of a census of cervical cytology; we show the distribution of cytology results, by age, in the general (screening) population and women with cervical cancer. We explore the sensitivity and positive predictive value (PPV) of cervical cytology at different thresholds for the detection of invasive cervical cancer in the general screening population, as well as the number of women needed to be screened (NNS) to detect one case of invasive cervical cancer.

During the study period (2006–2010) the technology used for screening in England changed. Liquid‐based cytology (LBC) was introduced between 2003 and 2008 and will have resulted in cytology slides taken during the study period reported in a combination of conventional cytology and one of the two systems of LBC. In addition, six large cytology laboratories in England were using HPV testing to triage borderline and mild dyskaryosis(equivalent to ASC‐US and LSIL, respectively, in the Bethesda system) during most of this period.

## Materials and methods

For the results of cytology in women with cervical cancer, we used data from the National Audit of Invasive Cervical Cancers in England.^1^, ^2^, ^3^ We studied a retrospective cohort of women who had had cervical cytology taken in the 12 months prior to diagnosis, between April 2007 and March 2010. All cervical cytology was read using the British Society for Clinical Cytology (BSCC) terminology in laboratories subject to accreditation and quality assurance. Data on their screening histories were abstracted from cervical cytology records held on the Exeter Call/Recall System. For results in the general population we used an extract from the Exeter database (taken in October/November 2010) including annual attendances to the screening programme from April 2007 to March 2010.^4^ This resulted in a census of cytology results. The information in this extract included the women's age, test result and category of screening invitation (for example routine recall, early recall after an abnormality or surveillance after treatment). In both women with cancer and the general population, we excluded cytology that was taken because of an earlier abnormal result. Note that by considering cytology in a 3‐year window and excluding repeat tests, few women will have more than one test in this study. For women with cervical cancer, we define the ‘index test’ as the first non‐recall (i.e. not a follow‐up test) test result within 12 months of diagnosis. Twelve months was chosen to allow for diagnosis after early (6‐month) recall triggered by borderline or mild dyskaryotic cytology (equivalent to ASC‐US and LSIL, respectively, in the Bethesda system) while ensuring that the cancer was already present at the time of cytology. Sensitivity analyses taking the index test as the first within 9 and 18 months of diagnosis were also carried out.

The BSCC terminology can be compared broadly with the Bethesda System (TBS) as follows^5^: Borderline changes (early recall) includes atypical squamous cells (ASC) and atypical glandular cells (AGC). Borderline, high‐grade dyskaryosis not excluded, equivalent to ASC, cannot exclude a high‐grade squamous intraepithelial lesion (ASC‐H). The term dyskaryosis equates to a squamous intraepithelial lesion (SIL); mild dyskaryosis corresponds to a low‐grade squamous intraepithelial lesion (LSIL); high‐grade dyskaryosis (equating to HSIL) is defined as moderate or severe dyskaryosis. Separate categories exist for severe dyskaryosis/?invasive and ?glandular neoplasia for squamous cell carcinoma and cervical glandular intraepithelial neoplasia (CGIN) ⁄ adenocarcinoma, respectively.

Borderline cytology results were divided into two groups reflecting the associated differing risk of disease. ‘Borderline‐high risk’ comprises tests for which immediate referral to colposcopy is recommended (because the high‐grade disease could not be ruled out). ‘Borderline low‐risk’ comprises tests for which a repeat test at 6 months is recommended.^6^ In England, although text reports distinguish borderline changes in which high‐grade cannot be excluded, result codes on national records do not record subtypes of borderline. Because we did not have access to cytology reports, we used cytology test action codes of ‘suspend’ (i.e. referral to colposcopy) and ‘early‐recall’ (i.e. repeat cytology in 6 months) to classify women as ‘borderline‐high risk’ and ‘borderline low‐risk’, respectively. Borderline samples with a positive HPV triage test and those with changes in endocervical cells are included in the borderline‐high risk group as these samples would have triggered an immediate referral to colposcopy. Cytology test action codes were available for all women with cancer and a random sample (i.e. controls from the National Audit) of the general population. The proportion of controls classified as ‘borderline‐high risk’ was applied to the census of cytology results to estimate the proportion of the general population.

We estimated the number needed to screen (NNS), the positive predictive value (PPV) and sensitivity for cervical cancer according to three different groupings of cytology test results. This allowed us to assess which women would be likely to benefit from a 2‐week referral to colposcopy for further investigation. These were defined as follows: Level 1 includes severe dyskaryosis or worse cytology results; Level 2 includes borderline‐high risk, moderate dyskaryosis or worse cytology results; and Level 3 includes all cytological tests that resulted in a referral to colposcopy based on the screening programme protocols prior to diagnosis. Box 1 details the BSCC and Bethesda terminology included in each referral threshold Level.

Box 1 **Referral threshold Levels^a^ for colposcopy with their respective BSCC and Bethesda terminology**

**Table:** 

Level 1		Level 2		Level 3	
BSCC	Bethesda	BSCC	Bethesda	BSCC	Bethesda
Severe dyskaryosis	HSIL	Borderline, high‐grade dyskaryosis not excluded	ASC‐H	Third consecutive inadequate test	Unsatisfactory for evaluation
Severe dyskaryosis ?invasive	Squamous cell carcinoma	Borderline change in endocervical cells	Atypical endocervical, endometrial or glandular.	First or second mild; second or third consecutive borderline	LSIL, Atypical squamous cells of undetermined significance (ASC‐US).
?Glandular neoplasia	AGC, favour neoplastic/AIS Adenocarcinoma: endocervical endometrial extrauterine not otherwise specified (NOS)	High‐grade dyskaryosis, moderate or severe	HSIL	Borderline, high‐grade dyskaryosis not excluded	ASC‐H and AGC
		Severe dyskaryosis ?invasive	Squamous cell carcinoma	High‐grade dyskaryosis, moderate or severe	HSIL
		?Glandular neoplasia	AGC, favour neoplastic/AIS	Severe dyskaryosis ?invasive	Squamous cell carcinoma
			Adenocarcinoma: endocervical endometrial extrauterine not otherwise specified (NOS)	?Glandular neoplasia	AGC, favour neoplastic/AIS Adenocarcinoma: endocervical endometrial extrauterine not otherwise specified (NOS)

a Note: threshold levels are not mutually exclusive. BSCC, British Society for Clinical Cytology; LSIL, Low‐grade Squamous Intraepithelial Lesion; HSIL, High‐grade Squamous Intraepithelial Lesion; AIS, adenocarcinoma *in situ*; ASC‐H, atypical cells of undetermined significance, cannot exclude a high‐grade squamous intraepithelial lesion; ASC‐US, atypical cells of undetermined significance; AGC, atypical glandular cells.

### Statistical analysis

Sensitivities were calculated as the proportion of women with cancer who had a positive test when considering each cytology result. Specificities were calculated as the proportion of women without cancer who had a test result less severe than the relevant cut‐off. The PPVs were calculated by dividing the number of cancers diagnosed with a given index test result by the number of cytology tests (in the general population during the same period) with that test result. It was not possible to calculate the PPV for Level 3 cytology as the action code of each cytology test was not available in our extract of the Exeter database. The NNS is the total number of cytology tests divided by the total number of cancers diagnosed after cytology with that result or worse. To calculate the 95% confidence intervals (CIs) for the NNS, a 95% CI for the PPV was calculated assuming the number of cancers diagnosed from the number of cytology tests carried out has a binomial distribution, and the inverse was taken.^7^ Confidence intervals for borderline‐low risk and borderline‐high‐risk took into account (by simulation) that the number of cytology tests in each of these categories in the general population was estimated. Analyses were carried out in STATA 12 (StataCorp., College Station, TX, USA).

## Results

### Cytology results

**Figure 1:**
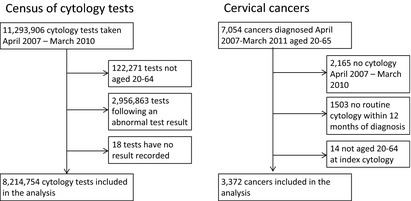
Inclusions and exclusions from the study

There were 3372 women diagnosed with cervical cancer, who had a routine (i.e. one not following a previous action code of early recall or suspend) cytology test within 12 months of diagnosis, between 1^st^ April 2007 and 31^st^ March 2010. Over the same period, there were 8 214 754 routine cytology tests in England (Figure 1). The majority of screening age cytology tests were negative (92.2%), 1.3% were Level 2 (moderate dyskaryosis or worse including borderline‐high risk), and 0.6% were Level 1 (severe dyskaryosis or worse) (Table 1). The percentage that was negative increased with age (Supplementary Table S1), from 82.0% in women aged 20–24 years to 94.9% in women aged 50–64 years. The proportion of tests that were inadequate remained consistent across age groups, ranging from 2.6% to 3.1% and remained similar over the study period. The percentage that were Level 2 was higher in young women (20–24 years: 3.7%, 25–34 years: 2.4%) than the older age groups (35–49 years: 0.9%, 50–64 years: 0.4%) (Table 2).

**Table 1:** Result of the first routine cytology test in the last 12 months in women aged 20–64 years, with cervical cancer (‘Cancers’) and in the general population (‘Cytology tests’) and the positive predictive value (PPV) of the test result to cervical cancer

Cytology test result^a^	Aged 20–64				
	Cancers	% cancers diagnosed with test result or worse	Cytology tests	% of all tests	PPV
Negative	106	100	7 570 265	92.2	0.00%
Inadequate	58	97	221 956	2.7	0.03%
Borderline – low risk	118	95	203 322	2.5	0.06%
Mild dyskaryosis	76	92	110 333	1.3	0.07%
Borderline – high risk	164	89	26 672	0.3	0.61%
Moderate dyskaryosis	215	85	32 823	0.4	0.66%
Severe dyskaryosis	1589	78	43 930	0.5	3.62%
?Glandular neoplasia	459	31	3701	0.0	12.40%
?Invasive squamous carcinoma	587	17	1752	0.0	33.50%
Total	3372		8 214 754		0.04%
Level 1	2635	78	49 383	0.6	5.34%
Additional value of moderate dyskaryosis and borderline‐high risk cytology	379	11	59 495	0.7	0.64%

a First routine cytology test in the last 12 months.

**Table 2:** Positive predictive value (PPV) of high grade (Level 2) and severe dyskaryosis or worse (Level 1) cytology to cervical cancer by age at diagnosis

Age group	Tests and cancers between April 07/March 10				
	Cancers with test result	% cancers diagnosed with test result	Cytology tests with result	% of all Cytology tests	PPV (%)
Level 2^a^					
20–24	97	87	9599	3.7	1.0
25–34	1246	91	57 006	2.4	2.2
35–49	1293	89	34 825	0.9	3.7
50–64	378	86	7447	0.4	5.1
Level 1^a^					
20–24	76	68	3785	1.5	2.0
25–34	1069	78	27 484	1.2	3.9
35–49	1160	80	14 936	0.4	7.8
50–64	330	75	3178	0.2	10.4

a First routine cytology test in the last 12 months.

### NNS, sensitivity and predictive values

**Figure 2:**
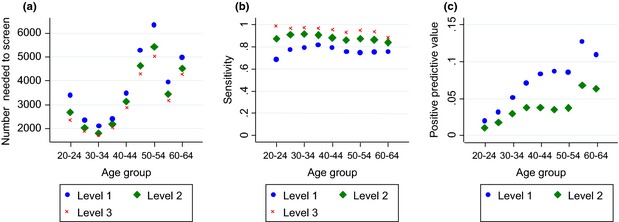
Number needed to screen, sensitivity and positive predictive value of cytology for cancer, by age group in three different Levels of cytology cut off. Level 1: severe dyskaryosis or worse; Level 2 borderline‐high‐risk or worse; and Level 3 referred to cytology as per screening programme protocols.

The NNS to identify one cancer in women aged 20–64 years with Level 2 cytology was 2726 (95% CI: 2630–2826) and the NNS was lowest for women aged 25–39 (1913, 95% CI: 1810, 2024). Trends in the NNS with age are shown in Figure 2a. The NNS is lowest when the incidence of cervical cancer is highest, at ages 25–39 years.

In 78% of women aged 20–64 years with cervical cancer with at least one test within 12 months of diagnosis, the first such test was Level 1 and 89% were Level 2. The sensitivity of cytology to cervical cancer was largely independent of age (Figure 2b).

Overall, the PPV for cervical cancer of a severe dyskaryosis/?invasive report was 33.5% (95% CI: 31.3–35.8%), the PPV of a report of ?glandular neoplasia was 12.4% (95% CI: 11.4–13.5%) and 3.6% (95% CI: 3.4–3.8%) for a report of severe dyskaryosis (Table 1). The PPV of moderate dyskaryosis was much less: 0.66% (95% CI: 0.57–0.75%) (i.e. 1 in 153 women with moderate dyskaryosis had cervical cancer), which was similar to the PPV of a borderline‐high risk (0.61%, 95% CI: 0.52–0.71%). The overall PPV of all Level 2 cytology was 2.77% (95% CI: 2.67–2.87%), whereas the PPV of Level 1 cytology was 5.34% (95% CI: 5.14–5.54%) (Table 1). The PPV of Levels 1 and 2 cytology increased with age (Table 2). The PPV of inadequate tests remained below the overall PPV for all women having a cytology test across all ages (0.03% for women aged 25–34 years, 35–49 years and 50–64 years). Trends in PPV of cytology to cancer by age are shown in Figure 2c.

The PPVs of mild dyskaryosis (0.07%, 95% CI: 0.05–0.09%) and of borderline‐low risk (0.06%, 95% CI: 0.05–0.07%) were similar to the prevalence of cancer in all screened women (0.041%, 95% CI: 0.040–0.042%). In other words, the chance of the cancer being diagnosed in the next year in a woman with mild dyskaryosis is only slightly higher than in a randomly selected woman attending screening (1 in 2436).

### Women's first screen

When data for young women were split according to whether it was the woman's first screen or subsequent screen, the proportion of Level 2 results was slightly higher for first screens (age 20–24 years: first screen 4.0%, subsequent screens 3.6%; age 25–29: first screen 3.2%, subsequent screens 2.7%). However, the PPV of a Level 1 cytology result was substantially higher for first screens than subsequent screens, particularly at age 20–24 years [first screen 4.51% (95% CI: 3.45–5.78%), subsequent screens 0.69% (95% CI: 0.40–1.10%)] (Table 3). This reflects the fact that prevalent occult cancers are most likely detected on the first screen.

**Table 3:** Predictive value of cytology to cervical cancer by age at diagnosis

Cytology test result*	Tests and cancers between April 07/March 10					Tests and cancers between April 07/March 10				
	Cancers	% cancers diagnosed with test result or worse	Cytology tests	% of all tests	PPV	Cancers	% cancers diagnosed with test result or worse	Cytology tests	% of all tests	PPV
	**Age 20–24 – first screen**					**Age 20–24 – subsequent screen**				
Negative	0	100	63 167	83.2	0.00%	0	100	148 763	81.5	0.00%
Inadequate	0	100	2029	2.7	0.00%	0	100	5480	3.0	0.00%
Borderline – low risk/mild dyskaryosis	7	100	7694	10.1	0.09%	7	100	21 693	11.9	0.03%
Borderline – high risk/moderate dyskaryosis	15	91	1698	2.2	0.88%	6	77	4116	2.3	0.15%
Severe dyskaryosis or worse	59	73	1307	1.7	4.51%	17	57	2478	1.4	0.69%
Total	81		75 895		0.107%	30		182 530		0.016%
	**Age 25–29 – first screen**					**Age 25–29 – subsequent screen**				
Negative	4	100	365 199	86.4	0.00%	13	100	743 270	88.0	0.00%
Inadequate	2	99	10 800	2.6	0.02%	5	97	21 423	2.5	0.02%
Borderline – low risk/mild dyskaryosis	10	98	33 370	7.9	0.03%	30	96	56 991	6.7	0.05%
Borderline – high risk/moderate dyskaryosis	28	94	7040	1.7	0.40%	65	89	12 071	1.4	0.54%
Severe dyskaryosis or worse	226	84	6330	1.5	3.57%	310	73	10 674	1.3	2.90%
Total	270		422 739		0.064%	423		844 429		0.050%
	**Age 30–34 – first screen**					**Age 30–34 – subsequent screen**				
Negative	2	100	100 522	89.2	0.00%	11	100	915 341	91.2	0.00%
Inadequate	5	98	3417	3.0	0.15%	4	98	26 843	2.7	0.01%
Borderline – low risk/mild dyskaryosis	3	94	6200	5.5	0.05%	31	97	43 336	4.3	0.07%
Borderline – high risk/moderate dyskaryosis	14	92	1249	1.1	1.12%	70	92	9163	0.9	0.76%
Severe dyskaryosis or worse	100	81	1308	1.2	7.65%	433	79	9172	0.9	4.72%
Total	124		112 696		0.110%	549		1 003 855		0.055%

### Results by stage at diagnosis

FIGO (International Federation of Gynaecologists and Obstetricians) stage was recorded for 91.6% of cancers diagnosed aged 20–64 years. Over half of those without cytology in the 12 months prior to diagnosis had stage 2+ (57.5%) compared to only 11.0% of women with cytology in the 12 months prior to diagnosis (Table 4).

**Table 4:** Screening category by FIGO stage in women aged 20–64 years

	Stage 1A cancers	% of cancers diagnosed at Stage 1A	Stage 1B cancers	% of cancers diagnosed at Stage 1B	Stage 2+ cancers	% of cancers diagnosed at Stage 2+
Not screened	93	9.0	345	33.5	593	57.5
Missed^a^	147	52.0	153	37.1	92	11.0
Cytology detected	1836		1262		326	
Total	2076		1760		1011	

a Has had a cytology test within a year of diagnosis, but no ‘suspend’ action code within a year of diagnosis.

The results were very similar when cytology within 9 or 18 months of diagnosis was considered in place of 12 months (Supplementary Table S2).

## Discussion

We have shown that in a screening population, regardless of age, the predictive value of cytology for cervical cancer is extremely high for reports of severe dyskaryosis/?invasive carcinoma (34%) and ?glandular neoplasia (12%), and high for reports of severe dyskaryosis (3.6%). Collectively these three groups account for a high proportion (78%) of invasive cervical cancers, and thereby confirm that the current guidelines for a 2‐week referral to colposcopy for severe dyskaryosis or worse cytology results are appropriate. Additionally, our results demonstrate the need for failsafe monitoring for women with severe dyskaryosis or worse cytology.

The PPV of severe dyskaryosis or worse (Level 1) cytology was substantially higher on the first cervical screening test in young women than among those who had been screened previously, reflecting the large number of prevalent cancers diagnosed at first screen, although it is important to note that in England 72% of women aged 20–29 years diagnosed with cervical cancer on their first cytology test had stage 1A cancer.^8^ These results explain why the number needed to screen is lower and the PPV of cytology to cancer is higher among women under the age of 35 years than among older women. The sensitivity of cytology for cervical cancer is excellent; in fact it is much better than the reported sensitivity for CIN3 or worse.^9^ The sensitivity of cytology to cancer was similar across all age groups.

Our results show that the risk of cancer in women aged 20–64 years with borderline low‐risk or mild dyskaryosis (ASCUS/LSIL) was similar (PPV of borderline‐low risk changes or mild dyskaryosis 0.06% and 0.07%, respectively) to that in all women tested (regardless of the result, 0.04%). In contrast, the PPVs of borderline‐high risk (0.61%) and moderate dyskaryosis (0.66%) were 15 times higher than the overall risk among screened women.

When interpreting these findings it is important to bear in mind that results refer to the use of cervical cytology for the early diagnosis of cancer, and not the standard use of cytology (i.e. screening for the detection of precursor disease). As such, the NNS will be much higher and predictive values in this paper much lower than those obtained in a screening context.

The main strength of this study is in the use of cytology results for the entire population of England together with the linked screening histories for women diagnosed with cervical cancer. Thus, it has wide‐ranging validity and is essentially unbiased. The number of borderline cytology tests that were high risk in the screening population had to be estimated from the audit dataset. However, as a result of the introduction of HPV triage in England, the new BSCC terminology guidelines^10^ no longer include details on whether or not high‐grade disease can be excluded from borderline results, relying instead on the HPV test result. Further, the introduction of LBC testing in England resulted in vigorous training for laboratory and primary care staff just prior to and during the study period, which may have affected the PPV of cytology.

Despite the fact that vaginal cytology was first described as a method of detecting cervical cancer^11^, we are not aware of any studies showing the PPV of modern cervical cytology to cancer. Results presented here demonstrate the usefulness of cytology with a threshold of borderline‐high risk or worse (Level 2) for the diagnosis of cervical cancer, regardless of age. In fact, these cytology results were observed in 89% of cancers.

There has been concern that cytology in the presence of invasive cancer is unreliable because the cytology may be inadequate (or even negative). In this study, we found no evidence (in any age group) to suggest this, and additionally show that the proportion of cytology tests that are inadequate in the presence of cancer remains low across all age groups. As some false‐negative cytology does occur, it remains important that GPs follow‐up women with gynaecological symptoms of possible cervical cancer, and that they make a specialist referral if symptoms persist or worsen.

A substantial benefit of cervical screening comes from early diagnosis of occult cancers. The NNS to detect one cancer using cervical cytology (2700 per cancer) is considerably higher than the NNS to diagnose one colorectal cancer using faecal occult blood tests (516 per cancer^12^). However, the incidence of cervical cancer has been reduced by 46% since the introduction of the national screening programme in England in 1988, and it is difficult to estimate what the incidence would be in the absence of screening.

This study reports cytology read using BSCC terminology in laboratories subject to accreditation and quality assurance, and the conclusions may not generalize to other systems for reporting cytology or to countries with less quality assurance.

## Conclusion

The PPV of severe dyskaryosis or worse cytology for cervical cancer is 4–10% in women aged 25–64 years, clearly justifying a 2‐week referral to colposcopy and emphasizing the importance of failsafe monitoring for such patients. The sensitivity of cytology for cervical cancer is excellent across all age groups.

## Ethical approval

The collection of data as part of audit has been covered since 2003 by section 251 of the NHS Act 2006 re‐enacted Section 60 of the Health and Social Care Act 2001 approval (PIAG 1‐08(a)/2003). The analysis of anonymised data in this context is considered service evaluation that does not require research ethics approval according to the UK guidelines (NRES).^13^

## Supporting information


**Table S1. **Result of the first non‐recall cytology test in the last 12 months by age group in women with cervical cancer and in the general population and predictive value of the test result to cervical cancer.
**Table S2. **Sensitivity analysis: result of the first non‐recall cytology test in the last 9 and 18 months in women aged 20–64 years with cervical cancer (‘Cancers’) and in the general population (‘Cytology tests’) and positive predictive value (PPV) of the test result to cervical cancer.

## References

[1] Sasieni P , Castanon A , Louie K . NHSCSP Audit of Invasive Cervical Cancer. National Report 2007‐2010: 2011.Available from: http://www.cancerscreening.nhs.uk/cervical/publications/nhscsp-audit-invasive-cervical-cancer-201107.html.

[2] Sasieni P , Castanon A , Cuzick J . Effectiveness of cervical screening with age: population based case‐control study of prospectively recorded data. Br Med J 2009;339:b2968. 19638651 10.1136/bmj.b2968 PMC2718082

[3] The NHS Information Centre Screening and Immunisations team . Cervical Screening Programme ‐ England, 2010–11: Report: 2011Available from: www.cancerscreening.nhs.uk/cervical/cervical-statistics-bulletin-2010-11.pdf.

[4] Lancucki L , Sasieni P , Patnick J , Day T , Vessey M . The impact of Jade Goody's diagnosis and death on the NHS Cervical Screening Programme. J Med Screen 2012;19:89–93. 22653575 10.1258/jms.2012.012028 PMC3385661

[5] Denton KJ , Herbert A , Turnbull LS *et al*, *et al* The revised BSCC terminology for abnormal cervical cytology. Cytopathology 2008;19:137–57. 18494998 10.1111/j.1365-2303.2008.00585.x

[6] National Cancer Screening Service . Guidelines for Quality Assurance in Cervical Screening: 2009 Available from: http://www.cancerscreening.ie/publications/QA_final_web_version.pdf.

[7] Altman DG . Confidence intervals for the number needed to treat. Br Med J 1998;317:1309. 9804726 10.1136/bmj.317.7168.1309 PMC1114210

[8] Castanon A , Leung V , Landy R , Lim A , Sasieni P . Characteristics and screening history of women diagnosed with cervical cancer aged 20‐29 years. Br J Cancer 2013;109:35–41. 23820257 10.1038/bjc.2013.322 PMC3708588

[9] Cuzick J , Clavel C , Petry KU *et al*, *et al* Overview of the European and North American studies on HPV testing in primary cervical cancer screening. Int J Cancer 2006;119:1095–101. 16586444 10.1002/ijc.21955

[10] Achievable standards, Benchmarks for reporting, and Criteria for evaluating cervical cytopathology (3rd edition). Editors:Smith J, Patnick J.NHSCSP publication No1. Sheffield: 2013 Available from: http://www.cancerscreening.nhs.uk/cervical/publications/nhscsp01.pdf

[11] Papanicolaou GN , Traut HF . Diagnosis of Uterine Cancer by the Vaginal Smear. New York: The Commonwealth Fund; 1943.

[12] Mansouri D , McMillan DC , Grant Y , Crighton EM , Horgan PG . The impact of age, sex and socioeconomic deprivation on outcomes in a colorectal cancer screening programme. PLoS ONE 2013;8:e66063. 23776606 10.1371/journal.pone.0066063 PMC3680425

[13] National Patient Safety Agency . NRES Leaflet: Defining research. 2009; Available from: http://www.nres.nhs.uk/applications/is-your-project-research/.

